# Clonal Candidemia Outbreak by *Candida parapsilosis* Carrying Y132F in Turkey: Evolution of a Persisting Challenge

**DOI:** 10.3389/fcimb.2021.676177

**Published:** 2021-04-22

**Authors:** Amir Arastehfar, Suleyha Hilmioğlu-Polat, Farnaz Daneshnia, Weihua Pan, Ahmed Hafez, Wenjie Fang, Wanqing Liao, Zümrüt Şahbudak-Bal, Dilek Yeşim Metin, João N. de Almeida Júnior, Macit Ilkit, David S. Perlin, Cornelia Lass-Flörl

**Affiliations:** ^1^ Center for Discovery and Innovation, Hackensack Meridian Health, Nutley, NJ, United States; ^2^ Division of Mycology, Department of Microbiology, Faculty of Medicine, University of Ege, Izmir, Turkey; ^3^ Shanghai Key Laboratory of Molecular Medical Mycology, Shanghai Institute of Mycology, Shanghai Changzheng Hospital, Second Military Medical University, Shanghai, China; ^4^ Biotechvana, Valencia, Spain; ^5^ Division of Pediatric Infectious Diseases, Ege University, Izmir, Turkey; ^6^ Laboratorio de Micologia Medica (LIM 53), Instituto de Medicina Tropical, Universidade de São Paulo, São Paulo, Brazil; ^7^ Laboratório Central (LIM 03), Hospital das Clínicas da Faculdade de Medicina da Universidade de São Paulo, São Paulo, Brazil; ^8^ Division of Mycology, Department of Microbiology, Faculty of Medicine, University of Çukurova, Adana, Turkey; ^9^ Institute of Hygiene and Medical Microbiology, Medical University of Innsbruck, Innsbruck, Austria

**Keywords:** antifungal, antifungal agent, molecular type, microdilution, fluconazole

## Abstract

As the second leading etiological agent of candidemia in Turkey and the cause of severe fluconazole-non-susceptible (FNS) clonal outbreaks, *Candida parapsilosis* emerged as a major health threat at Ege University Hospital (EUH). Evaluation of microbiological and pertinent clinical profiles of candidemia patients due to *C*. *parapsilosis* in EUH in 2019–2020. *Candida parapsilosis* isolates were collected from blood samples and identified by sequencing internal transcribed spacer ribosomal DNA. Antifungal susceptibility testing was performed in accordance with CLSI M60 protocol and *ERG11* and HS1/HS2-*FKS1* were sequenced to explore the fluconazole and echinocandin resistance, respectively. Isolates were typed using a multilocus microsatellite typing assay. Relevant clinical data were obtained for patients recruited in the current study. FNS *C*. *parapsilosis* isolates were recovered from 53% of the patients admitted to EUH in 2019–2020. Y132F was the most frequent mutation in Erg11. All patients infected with *C*. *parapsilosis* isolates carrying Y132F, who received fluconazole showed therapeutic failure and significantly had a higher mortality than those infected with other FNS and susceptible isolates (50% *vs*. 16.1%). All isolates carrying Y132F grouped into one major cluster and mainly recovered from patients admitted to chest diseases and pediatric surgery wards. The unprecedented increase in the number of Y132F *C*. *parapsilosis*, which corresponded with increased rates of fluconazole therapeutic failure and mortality, is worrisome and highlights the urgency for strict infection control strategies, antifungal stewardship, and environmental screening in EUH.

## Introduction

Candidemia represents a major global public health concern due to high mortality rates and additional economic burden (Arastehfar et al., 2020f). Although global epidemiological studies characterize *Candida albicans* as the leading cause of candidemia (Pfaller et al., 2019), non-*albicans Candida* (NAC) species, especially *C*. *parapsilosis*, *C*. *tropicalis*, *C*. *glabrata*, and *C*. *auris*, surpass *C*. *albicans* in some countries and individual patient population and healthcare institutions (Arastehfar et al., 2020f; Megri et al., 2020). Among these NAC species, *C*. *parapsilosis* stands out as an important causative agent of invasive candidiasis observed in the last decade (Pfaller et al., 2019). *Candida parapsilosis* is thought to be transferred through direct contact usually involving the hands of healthcare workers (Tóth et al., 2019). Its ability to form tenacious biofilms allows this species to persist avidly in the hospital settings (Tóth et al., 2019). Furthermore, numerous studies from European (Arastehfar et al., 2020b; Martini et al., 2020), Asian (Singh et al., 2019), African (Magobo et al., 2020), and Latin American (Thomaz et al., 2018) countries have reported an unprecedented rate of fluconazole and/or azole resistance due to *C*. *parapsilosis*, which, combined with the mode of transmission, has led to severe clonal outbreaks and establishment of persistent fluconazole-non-susceptible (FNS) *C*. *parapsilosis* in previously unknown niches of the hospital environment. Horizontal transmission results in fluconazole-resistant *C*. *parapsilosis* isolated from azole-naïve patients (Arastehfar et al., 2020b) and subsequent therapeutic failure, which can potentially lead to higher hospital expenses, longer hospitalization times, and poor outcome. To further complicate the matter, the emergence of multidrug-resistant (MDR) *C*. *parapsilosis* blood isolates showing resistance to both azoles and echinocandins has recently been reported (Arastehfar et al., 2020c; Arastehfar et al., 2021). Therefore, it is highly important to diligently monitor the burden of antifungal resistance and perform genotyping of *C*. *parapsilosis* blood isolates in clinical settings that have experienced fungal infection outbreaks.

A principal molecular mechanism underlying fluconazole resistance in *Candida* species involves mutations in the *ERG11* gene encoding an enzyme of the ergosterol biosynthetic pathway (Arastehfar et al., 2020f). Residue substitutions Y132F, K143R, and G458S in Erg11 are considered the major cause of fluconazole resistance, followed by the upregulation of *ERG11* and efflux pump genes *MDR1* and *CDR1* due to gain-of-function mutations in the respective transcription factors (Arastehfar et al., 2020f). Resistance to echinocandins develops through acquisition of mutations in hotspot (HS) regions of *FKS* genes (Arastehfar et al., 2020f; Arastehfar et al., 2021).

In our previous studies, we analyzed *C*. *parapsilosis* blood isolates recovered from Ege University Hospital (EUH) between 2007 and early 2019, and have reported severe clonal outbreaks due to fluconazole-resistant (Arastehfar et al., 2020b) and MDR (Arastehfar et al., 2021) *C*. *parapsilosis* isolates. The increasing trend of fluconazole-resistant *C*. *parapsilosis* blood isolates in EUH, especially from 2018 onward, and the lack of detailed clinical data with respect to fluconazole therapeutic failure (FTF) among infected patients from our previous studies prompted us to collect the blood isolates obtained in 2019 to 2020. Furthermore, we applied a multilocus microsatellite typing technique to assess the genetic relatedness of the *C*. *parapsilosis* blood isolates collected and to identify the wards, where clonal outbreaks are ongoing.

## Materials and Methods

This was a retrospective study, which included patients with candidemia due to *C*. *parapsilosis* admitted to EUH between January 2019 and January 2020. There were no exclusion criteria regarding age, sex, underlying conditions, wards, etc. Persistent fever and obtaining *C*. *parapsilosis* from blood culture despite antifungal treatment were considered as antifungal therapeutic failure and reported by treating physicians. Drug exposure included the recorded data of the preceding six months and if needed records of the previous hospitalizations were checked. Thirty-day mortality was defined when death occurred ≤ 30 days after the first positive blood culture (Kord et al., 2020). *Candida parapsilosis* blood isolates were inoculated onto Sabouraud glucose agar (Merck, Darmstadt, Germany) and chromogenic agar (Candi*Select*™ 4, Bio-Rad, Hercules, CA, USA) plates and incubated at 37°C for 24–48 h. Species identification was performed using primers targeting internal transcribed spacer 1 (ITS1; TCCGTAGGTGAACCTGCGG) and ITS 4 (GCATATCAATAAGCGGA) (Stielow et al., 2015). It has been shown that ITS1 and ITS4 can discriminate species within *C*. *parapsilosis* species complex (Souza et al., 2012).

### Antifungal Susceptibility Testing

Antifungal susceptibility testing was performed by the broth microdilution method according to the CLSI M60 protocol (CLSI, 2017) and included fluconazole, voriconazole, itraconazole, posaconazole, amphotericin B (all from Sigma-Aldrich, St. Louis, MO, USA), caspofungin (bioMérieux SA, Marcy-l’Étoile, France), micafungin (Astellas, Munich, Germany), and anidulafungin (Pfizer, New York, NYC, USA). Isolates were incubated at 35°C for 24 h and minimum inhibitory concentrations (MICs) were visually recorded. *Candida krusei* (ATCC 6258) and *C*. *parapsilosis* (ATCC 22019) were used for quality control. Resistance to fluconazole, anidulafungin, micafungin, and caspofungin was considered at MICs ≥ 8 μg/mL, whereas that to voriconazole was noted at MICs ≥ 1 μg/mL (Pfaller and Diekema, 2012). Isolates with the fluconazole MICs ≥4 μg/mL were regarded as FNS. Susceptibility to amphotericin B, itraconazole, and posaconazole was reported based on epidemiological cut-off values and isolates showing MICs >2, >0.5, and >0.25 μg/mL were considered non-wild-type (NWT) (Pfaller and Diekema, 2012).

Fluconazole tolerance was defined as incomplete growth inhibition at supra-MICs for 48 h compared to positive control (Arastehfar et al., 2020e; Berman and Krysan, 2020).

### DNA Extraction and Sequencing of ERG11 and FKS1

DNA was extracted using a previously described CTAB-based protocol (Arastehfar et al., 2018). ERG11 and HS1 and HS2 of *FKS1* were amplified by PCR using specific primers and sequenced as previously described (Arastehfar et al., 2019b; Arastehfar et al., 2020c). Since echinocandin-susceptible *C*. *parapsilosis* may occasionally carry mutations in the HS regions (Arastehfar et al., 2020d) and isolates with the susceptible dose-dependent phenotype for fluconazole may have the Y132F mutation (Singh et al., 2019), sequencing was performed for all isolates to detect any potential genetic changes conferring resistance and/or therapeutic failure.

Raw sequence data were edited using SeqMan Pro software (DNASTAR, Madison, WI, USA), and the curated sequences were aligned against WT sequences of *ERG11* (GQ302972) and *FKS* (Garcia-Effron et al., 2008) using MEGA v7.0 (Temple University, Philadelphia, PA, USA).

### Genotyping

The genetic relatedness of *C*. *parapsilosis* strains was evaluated using multilocus microsatellite typing targeting four loci and eight alleles (Trobajo-Sanmartín et al., 2018). Genotyping analysis was performed using BioNumerics software V7.6 (Applied Math Inc., Austin, Texas, USA) and different genotypes were assigned when two given strains differed in more than one allele. Genotypes showing with similar genetic profile were regarded as cluster.

### Statistical Analysis

The association between mortality and fluconazole susceptibility profile was assessed by chi-square test using SPSS v24 (SPSS Inc., Chicago, IL, USA). Kaplan-Meier survival curve was plotted using the GraphPad Prism software (GraphPad, San Diego, CA, USA). *P*-values below 0.05 were considered statistically significant.

### Deposition of the Generated Sequence Data

The sequences of *ERG11*, *FKS1*-HS1, *FKS1*-HS2 obtained in this study were deposited in the GenBank database under the following accession numbers (MW013584–MW013641), (MW013700–MW013757), and (MW013642–MW013699), respectively.

## Results

### Clinical Data

Between 2019 and 2020, 58 C. *parapsilosis* isolates were recovered from bloodstream of 47 patients. Multiple *C*. *parapsilosis* isolates were recovered from five patients: three with four and two – with two sequentially obtained isolates. The median age of the patients was 45 years (range, 40 days–89 years) and the majority of them were men (33/47; 70.2%). The most notable underlying conditions (some patients had more than one) were: cancer (12/47; 25.5%), surgery (10/47; 21.2%), burns (10/47; 21.2%), chronic respiratory diseases (6/47; 12.7%), diabetes mellitus (5/47; 10.6%), hypertension (5/47; 10.6%), neurological diseases (5/47; 10.6%), and cardiovascular diseases (4/47; 8.5%) (details in **Supplementary Table 1**). Central venous catheter was used in 63.8% of patients (30/47) and approximately 40.5% of those patients were admitted in intensive care units (ICUs) (19/47). Twelve patients (25.5%) received prophylactic treatment with azoles, including fluconazole (11/47; 23.4%) and posaconazole (1/47; 2.1%). Fluconazole (20/47; 42.5%), and echinocandins micafungin (15/47; 31.9%), caspofungin (12/47: 25.5%), anidulafungin (8/47; 17%), and amphotericin B (11/47; 23.4%) were used as targeted treatment. The overall mortality rate was 27.6%.

### Antifungal Susceptibility Profiles

All *C*. *parapsilosis* isolates were susceptible to echinocandins (anidulafungin, micafungin, and caspofungin) and were WT to amphotericin B, itraconazole, and posaconazole. The FNS phenotype (MICs ≥4 μg/mL) was detected in 28 isolates (MIC = 4 μg/mL, *n* = 12; MIC ≥ 8 μg/mL, *n* = 16) collected from 53% of patients (25/47) (**Tables 1** and **2**). Voriconazole resistance (MICs ≥1 μg/mL) and intermediate phenotypes (MIC = 0.25–0.5 μg/mL) were observed for seven and four isolates, respectively. Of note, the seven voriconazole-resistant isolates were cross-resistant to fluconazole (**Tables 1** and **2**).

**Table 1:** Comprehensive microbiological and clinical data of candidemic patients infected with *C*. *parapsilosis* in Ege University Hospital between 2019 to 2020.

Genotype	Ward (patient #)	Isolate #	MIC values (µg/mL)		Erg11p	Azole prophylaxis/empiric therapy	ATF/ATT	Mortality rate
			FLZ	VRZ				
**Fluconazole-non-susceptible isolates containing Y132F mutation**								
G19 (*n*= 1), G21 (*n*= 1), G22 (*n*= 3), G28 (*n*= 1)	Chest diseases (*n*= 6)	*n*= 17	4–32	0.032–0.5	Y132F	FLC (*n*= 4)	FLC (*n*= 11)/MFG (*n*= 4), AND (*n*= 3), CSP (*n*= 2), and LAMB (n= 2)	50% (8/16)
G19 (n= 2), G20 (*n*= 1), G21 (*n*= 1), G24 (*n*= 1)	Pediatric surgery (*n*= 5)**^A^**							
G23 (*n*= 1), G25 (*n*= 1)	Pediatric ICU (*n*= 2)							
G26 (*n*= 1)	Internal medicine (*n*= 1)							
G22 (*n*= 1)	Cardiovascular surgery (*n*= 1)						Azole-naïve (*n*= 5)/CSP (*n*= 2), LAMB (*n*= 2), not treated (*n*= 1)	
G31 (*n*= 1)	ICU burn (*n*= 1)							
C8/G27 (*n*= 3)	Pediatric (*n*= 2), Pediatric ICU (*n*= 1)	*n*= 3	>64	1–2	Y132F+G307A	FLC (*n*= 1)	FLC (*n*= 2)/MFG (*n*=1), LAMB (*n*= 1) Azole-naïve (*n*= 1)/CSP (*n*=1)	0% (0/3)
**Other mutations causing fluconazole resistance G458S**								
G13 (*n*= 2)	Pediatric surgery (*n*= 2)	*n*= 4	16–>64	0.25–2	G458S	FLC (*n*= 2)	FLC (*n*= 3)/MFG (*n*= 1), CSP (*n*= 1), LAMB (*n*= 1) Azole-naïve (*n*= 1)/AND (*n*= 1)	0% (0/4)
G13 (*n*= 1)	Pediatric gastroenterology (*n*= 1)							
G7 (*n*= 1)	Pediatric ICU (*n*= 1)							
G4 (*n*= 1)	Pediatric (*n*= 1)	*n*= 1	>64	4	G307A+G458S	No	FLC (*n*= 1)	0% (0/1)
**Other fluconazole-non-susceptible isolates without mutations conferring resistance**								
G1 (*n*= 3)	Burn ICU (*n*= 1)**^B^**	*n*= 3	4–8	0.064–0.125	R398I (n=3)	No	Azole-naïve/MFG and LAMB	0% (0/1)
**Fluconazole susceptible isolates**								
G1 (*n*= 1), G3 (*n*= 2), G6 (*n*= 1), G9 (*n*= 1), G11 (*n*= 1), G12 (*n*= 1), G16 (*n*= 2)	Burn ICU (*n*= 8)^B*^	*n*= 9^B*^	0.25–2	0.32–0.64	R398I (*n*= 6)	FLC (*n*= 4), POSA (*n*= 1)	FLC (*n*= 2)	22.7% (5/22)
G5 (*n*= 1), G15 (*n*= 4)	Pediatric (*n*= 2)	*n*= 5	0.5	0.32				
G14 (*n*= 1), G18 (*n*= 1)	Chest diseases (*n*= 2)	*n*= 2	0.25–2	0.32–0.64				
G10 (*n*= 1), G17 (*n*= 1)	Neurosurgery services (*n*= 2)	*n*= 2	0.5–1	0.32				
G5 (*n*= 1), G6 (*n*= 1), G7 (*n*= 1), G8 (*n*= 1)	Cardiovascular surgery (*n*= 1)	*n*= 4	0.5	0.32				
G6	Pediatric surgery (*n*= 1)^A*^	*n*= 1^A*^	0.5	0.32				
G6	Cardiology service (*n*= 1)	*n*= 1	0.5	0.32				
G6	Pediatric ICU (*n*= 1)	*n*= 1	0.5	0.32				
G30	General surgery (*n*= 1)	*n*= 1	0.25	0.32				
G6	Urology service (*n*= 1)	*n*= 1	0.5	0.32				
G2	Ear-nose-throat (*n*= 1)	*n*= 1	0.25	0.32				
G29	Infectious diseases (*n*= 1)	*n*= 1	0.5	0.32				
G6	Cardiovascular surgery ICU (*n*= 1)	*n*= 1	0.5	0.32				

**^A^** Two patients had duplicate isolates: the first (PA) was infected with two Y132F-carrying isolates and the second (PB) – with one fluconazole-susceptible WT isolate (denoted as ^A*^ and not included in the assessment of the mortality rate) and one fluconazole-non-susceptible Y132F-carrying isolate. **^B^** This patient was infected with four isolates: one WT, one fluconazole-susceptible (denoted as ^B*^ among fluconazole-susceptible isolates), and two fluconazole-resistant isolates not carrying any ERG11 mutations. Abbreviations: ATF, Aazole therapeutic failure; ATT, Alternative targeted treatment; FLC, Fluconazole; POSA, Posaconazole; CSP, Caspofungin; AND, Anidulafungin; MFG, Micafungin.

**Table 2:** Antifungal susceptibility data for *Candida parapsilosis* blood isolates collected from Ege University Hospital, Turkey, 2019–2020.

Antifungaldrugs	Minimum inhibitory concentration values (µg/ml)													Range		GM		MIC50		MIC90	
	0.03	0.06	0.125	0.25	0.5	1	2	4	8	16	32	≥64									
**Micafungin**					23	31	4							0.5–2		0.79		1		2	
**Anidulafungin**					6	46	6							0.5–2		1		1		2	
**Caspofungin**					22	32	4							0.5–2		0.80		1		1–2	
**Amphotericin B**				1	55	2								0.25–1		0.50		0.5		0.5	
**Fluconazole**				6	20	1	3	12	6	2	2	6		0.25–>64		2.22		2		32–64	
**Voriconazole**	31	7	9	2	2	3	3	1						0.03–4		0.07		0.03		1	
**Itraconazole**	44	3	5	6										0.03–0.25		0.04		0.03		1–2	
**Posaconazole**	42	12	3	1										0.03–0.25		0.03		0.03		0.06–0.125	

GM, Geometric mean; MIC, Minimum inhibitory concentration value.

### *ERG11* and *FKS1* Sequencing Results

**Figure 1:**
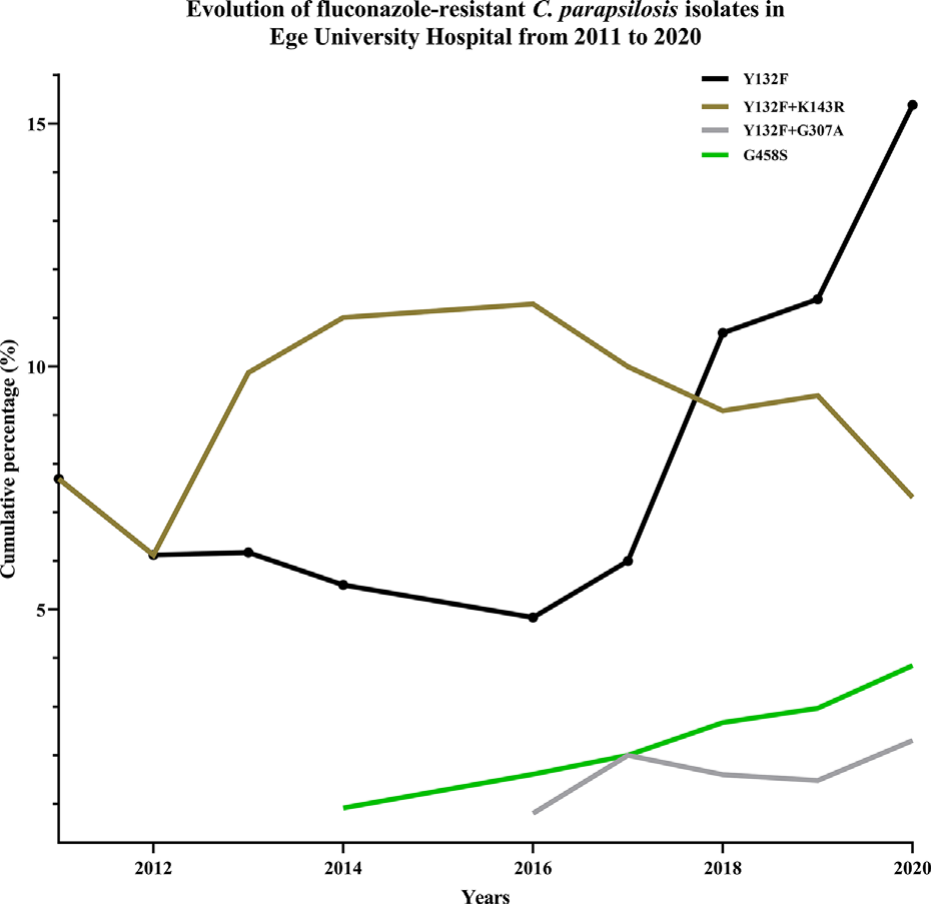
Mutation frequency and types in Erg11 showed a dynamic trend, where new mutations replaced previously dominant ones.

Consistent with the echinocandin MIC data, none of the *C*. *parapsilosis* isolates carried any mutations in HS1 or HS2 of *FKS1*. Among fluconazole resistance-related mutations, Y132F was the most prevalent (17/28; 60.7%), followed by G458S (4/28; 14.2%), Y132F+G307A (3/28; 10.7%), and G307A+G458S (1/28; 3.6%) (**Tables 1** and **2**). Interestingly, three FNS isolates recovered from a patient without any exposure to azoles during hospitalization did not carry any mutations in *ERG11* (**Tables 1** and **2**). In order to evaluate the dynamics of *ERG11* mutations in FNS *C*. *parapsilosis* isolates during the period between 2007 and 2020, we combined the current data with those obtained in our previous study (Arastehfar et al., 2020b) (**Figure 1**). Interestingly, we observed time-dependent shifts in the mutation type and frequency: the number of Y132F-carrying isolates was significantly increased from 2017 onward, whereas isolates with Y132F+K143R and K143R, which were prevalent before 2019, were not detected thereafter (**Figure 1** and **Table 1**). Importantly, isolates with newly emerged mutations (G458S and Y132F+G307A) also showed an increase from 2017 onward and one fluconazole-resistant isolate had a new mutation (G307A+G458S) not detected in our previous studies (**Figure 1** and **Table 1**). However, we did not observe any patients infected with clonal MDR *C*. *parapsilosis* isolates carrying Y132F+K143R in Erg11 or R658G in HS1 of Fks1, which is in contrast with our previous results (Arastehfar et al., 2021).

**Figure 2:**
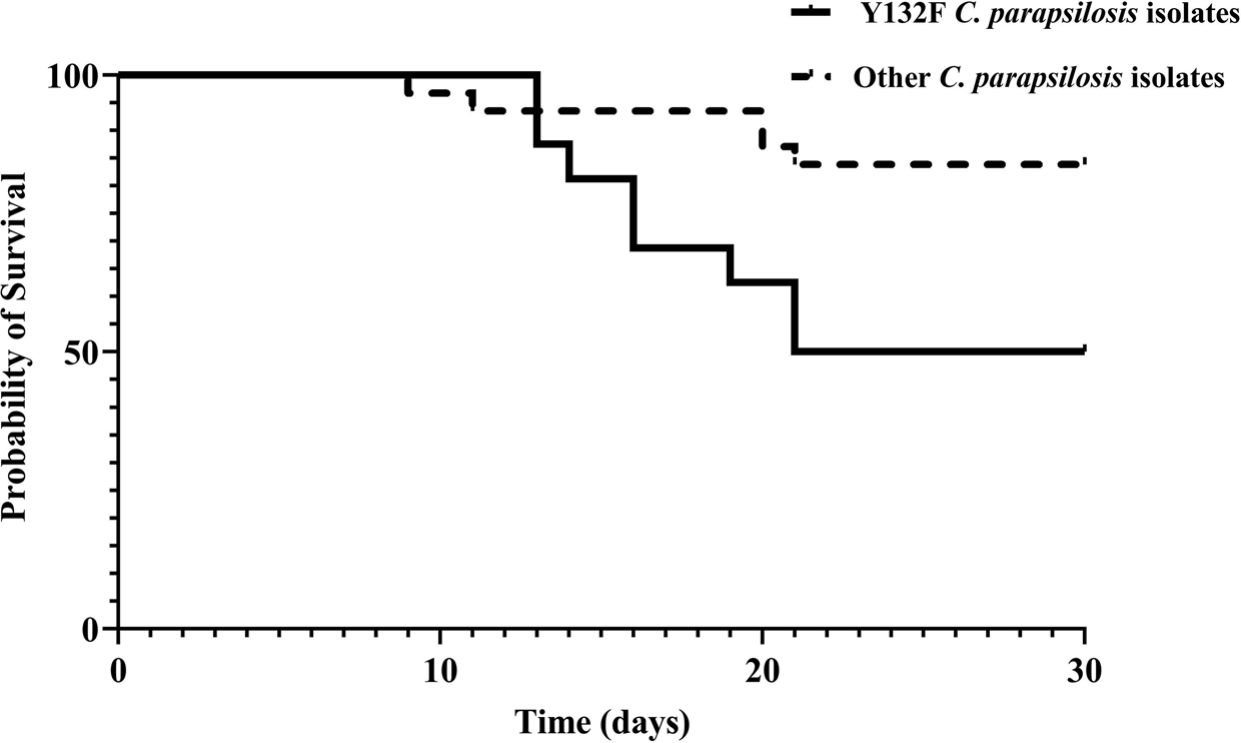
30-day survival of patients infected with azole-susceptible and azole-resistant *C*. *parapsilosis* isolates carrying Y132F in Erg11.

To assess the association between the emergence of FNS isolates and the use of antifungals, we analyzed the treatment records of the patients. All patients infected with FNS isolates and treated with fluconazole (17/25, 68%) showed FTF, while the rest of 8 patients carrying FNS *C*. *parapsilosis* isolates were azole-naïve (**Tables 1** and **2**). Patients infected with Y132F-carrying *C*. *parapsilosis* isolates significantly had a higher mortality rate (8/16; 50%) compared to those infected with other FNS and FS (5/31; 16.1%) isolates (**Tables 1** and **2**; **Figure 2**) (Chi-square, two-tailed, *P*= 0.012). FTF was also observed for two patients infected with eight FS isolates (four isolates per patient), but since there was no increase in MICs compared to positive control after 48 h, these isolates were not considered fluconazole-tolerant.

### Genotypic Diversity of *C. parapsilosis* Isolates

**Figure 3:**
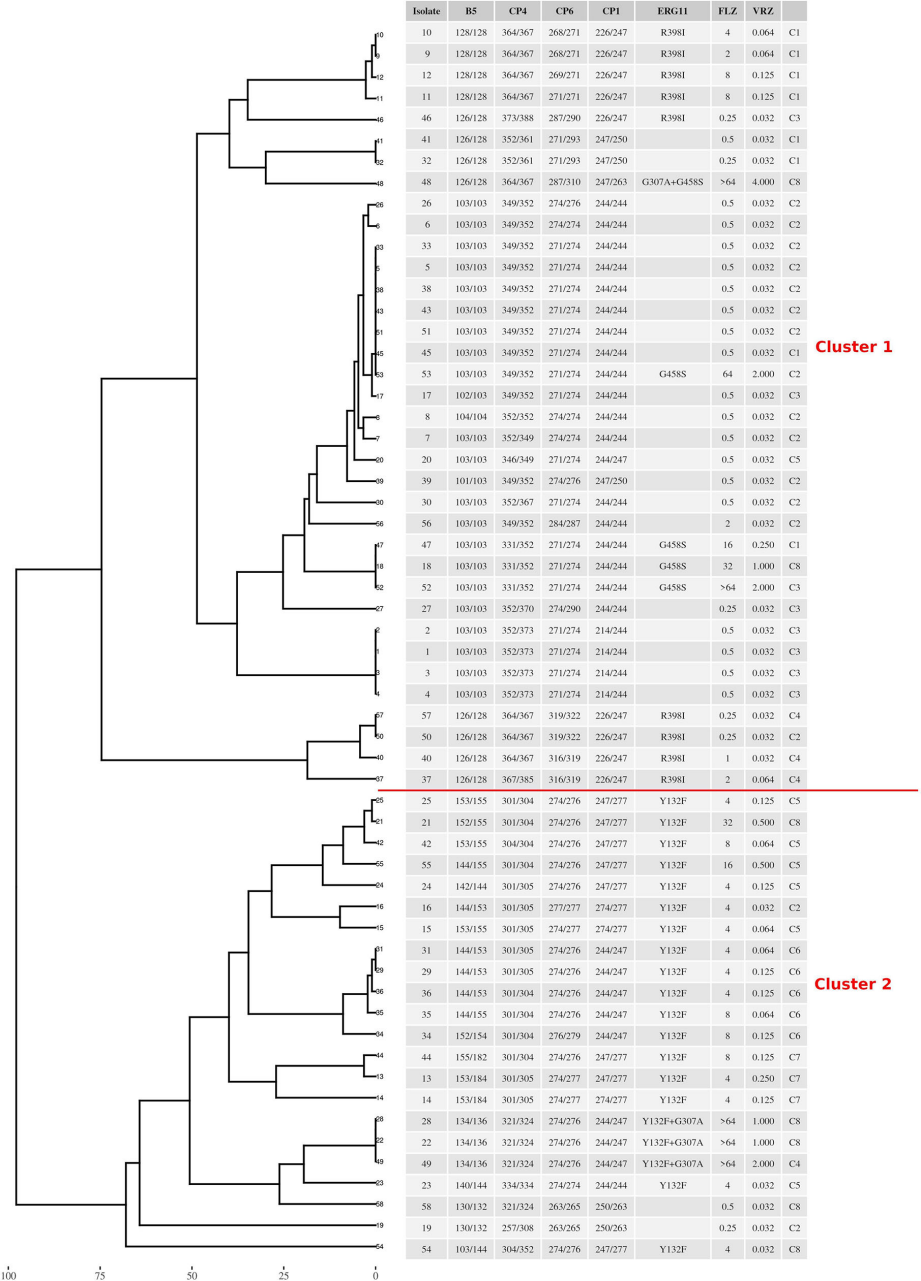
The genotypic relatedness of *C*. *parapsilosis* blood isolates recovered during 2019 to 2020 in Ege University Hospital reveals clonal outbreak due to both azole-susceptible and azole-non-susceptible isolates. C denotes minor cluster containing similar genotypes (difference ≥ 2 alleles).

**Figure 4:**
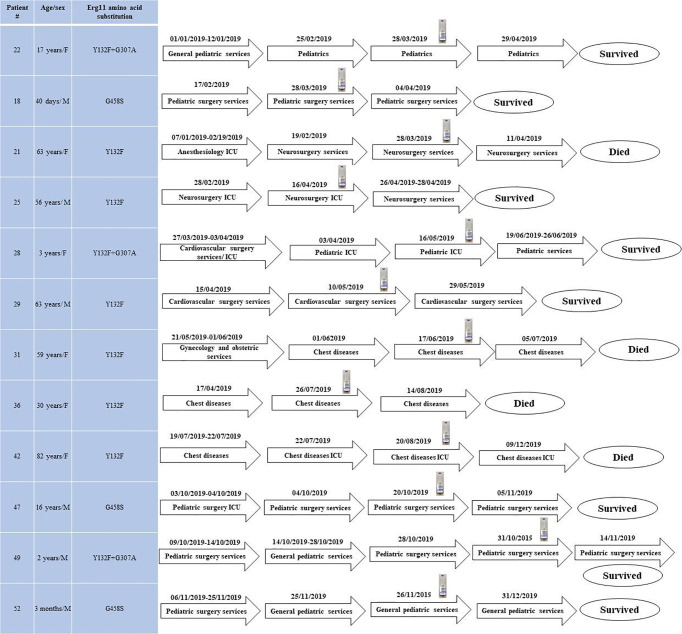
The transmission map of patients infected with fluconazole non-susceptible clonal isolates and/or with those showing high genetic similarity during the study period. Blood bottle symbol denotes the positive blood bottle date.

According to multilocus microsatellite typing, *C*. *parapsilosis* isolates were distributed into 2 major clusters, 8 sub-clusters (minor clusters), and 31 genotypes (**Figure 2** and **Table 1**). Interestingly, all isolates carrying Y132F or Y132F+G307A grouped into one major cluster and belonged to related genotypes, within which they formed small clonal clusters (**Figure 3** and **Table 1**). Approximately 69% of the patients infected with Y132F *C*. *parapsilosis* (11/16) were admitted to the chest and pediatric surgery wards, and those infected with Y132F+G307A isolates, which were 100% clonal, were admitted to pediatric wards and pediatric ICU (**Figure 3** and **Table 1**). The second major cluster comprised almost 91% of FS isolates (20/22) and some FNS isolates carrying G458S and G307A+G458S mutations (**Figure 3**, **Table 1**). This cluster contained two sub-clusters of clonal isolates including: FS isolates from seven patients (patients #5, 33, 38, 43, 45, 51, and 53), mainly obtained in burn ICU, and G458S-carrying isolates from three patients of pediatric surgery and gastroenterology wards (patients # 18, 47, and 52), respectively (**Figure 3** and **Table 1**). Details regarding the hospitalization of patients infected with FNS isolates with highly genetic similar profiles and/or clonal are shown in a transmission map in **Figure 4**. Collectively, these data indicate a continuing outbreak due to both FNS isolates with similar genetic profiles and clonal FS *C*. *parapsilosis* in various wards of EUH.

## Discussion

The emergence of clonal outbreaks due to FNS and MDR *C*. *parapsilosis* in EUH is a matter of clinical concern. Therefore, continuous monitoring of the burden of antifungal resistance as well as analysis of the underlying molecular mechanisms and genotypic diversity of the isolates in relation to clinical data are of paramount importance. An alarming finding is that approximately 53% of patients with candidemia in this study were infected with FNS *C*. *parapsilosis* and all of them showed FTF when treated with azoles. We observed a dynamic shift in the mutation type and frequency among FNS *C*. *parapsilosis* isolates, when some previously prevalent mutations disappeared and were replaced by new ones. Beyond FTF, the Y132F-carrying *C*. *parapsilosis* isolates were associated with the highest mortality rate compared to patients infected with other isolates.

Overall, the 53% of the patients infected with FNS *C*. *parapsilosis* represented 48% of all analyzed isolates. Moreover, almost 15% of the FNS isolates were also cross-resistant to voriconazole. Analysis of the burden of FNS isolates in EUH in 2007–2020 based on the combined results of this and the previous study (Arastehfar et al., 2020b) revealed its highest rate in 2019. An increased number of studies report a high rate of FNS *C*. *parapsilosis* blood isolates, which ranges from 22% and 32% in Italy and India (Singh et al., 2019; Martini et al., 2020) to 71% and 78% in Brazil and South Africa (Thomaz et al., 2018; Magobo et al., 2020). Consistent with these data, a recent global study showed increasing prevalence of FNS *C*. *parapsilosis* in Latin American countries (Pfaller et al., 2019). In contrast to our earlier study (Arastehfar et al., 2021) but in line with the global trend, in this study we did not detect any isolates showing resistance against echinocandins or the NWT phenotype to amphotericin B. Unfortunately, most reports on the clonal expansion of FNS *C*. *parapsilosis* are from developing countries, where high cost of echinocandins promotes the popularity of fluconazole as the main treatment of invasive *Candida* infections (Arastehfar et al., 2019a; Arastehfar et al., 2019c; Arastehfar et al., 2020a; Megri et al., 2020) which can undermine the clinical efficacy of fluconazole and may have detrimental consequences. Altogether, these data point to the danger of the increasing burden of FNS *C*. *parapsilosis* blood isolates in clinical centers.

To analyze the molecular mechanisms of antifungal resistance in our isolates, we sequenced *ERG11* and *FKS1* genes. In agreement with the drug susceptibility profiles, we did not identify any mutations in *FKS1* HS1 and HS2 but found numerous mutations in *ERG11* of FNS isolates, which showed dynamic changes over time. Interestingly, in contrast to our previous study indicating that Y132F+K143R was one of the most prevalent residue substitutions in Erg11p (Arastehfar et al., 2020b), here we did not find any isolates carrying Y132F+K143R or K143R, Q250K+R398I+G458S, and G458S+T519A, which were replaced by the emerging mutations Y132F+G307A, G458S, and G307A+G458S. Y132F was found in almost 60% of the current FNS isolates and its prevalence was higher in this study (2019–2020) compared to our previous study (2007–2019) (Arastehfar et al., 2020b). Our previous analysis using a large cohort of patients infected with *C*. *parapsilosis* showed that azole use in the hospital was higher from 2015 onward, which was speculated to be the cause behind this surge (Arastehfar et al., 2020b). Moreover, environmental contamination could be another factor further contributing in continuous outbreak and increasing number patients infected with *C*. *parapsilosis* isolates carrying Y132F isolates. Our data confirming Y132F as the leading mutation underlying azole resistance is consistent with reports from India (Singh et al., 2019), Brazil (Thomaz et al., 2018), South Korea (Choi et al., 2018), South Africa (Magobo et al., 2020), Italy (Martini et al., 2020), the United States (Grossman et al., 2015), and Kuwait (Asadzadeh et al., 2017), where other Erg11p mutations have rarely been identified, except for K143R detected in India (Singh et al., 2019). On the other hand, the extreme variety of *ERG11* mutations observed in our settings compared to the other studies (Grossman et al., 2015; Asadzadeh et al., 2017; Choi et al., 2018; Thomaz et al., 2018; Singh et al., 2019; Magobo et al., 2020; Martini et al., 2020) may highlight potential hypermutability of Turkish *C*. *parapsilosis* isolates and the presence of certain genetic mechanisms promoting the establishment and persistence of such outstanding genetic diversity.

To evaluate the clinical significance of our molecular data, we analyzed their association with patient outcome, specifically focusing on mortality and FTF. The overall mortality rate was 27.6%; however, it was significantly higher for patients infected with Y132F-carrying *C*. *parapsilosis* than for those infected with the other FNS and FS isolates, which is consistent with the trend reported in our previous study (Arastehfar et al., 2020b; Thomaz et al., 2021). Unfortunately, the retrospective nature of our study did not allow us to obtain detailed clinical data on the severity of the diseases, which directly impacts the mortality rate. Among other host- and drug-related factors involved, this phenomenon may be attributed to the presence of some unknown compensatory genetic mutations accompanying Y132F and/or higher virulence of Y132F strains compared to other isolates, which, consequently, may explain the exponential increase in the incidence of Y132F-carrying *C*. *parapsilosis* observed in our and other studies (Grossman et al., 2015; Asadzadeh et al., 2017; Choi et al., 2018; Thomaz et al., 2018; Singh et al., 2019; Arastehfar et al., 2020b; Magobo et al., 2020; Martini et al., 2020). Of note, our most recent study involving *Galleria mellonella* have shown the lack of a higher virulence attributes of *C*. *parapsilosis* isolates carrying Y132F compared to other FNS and FS isolates (Binder et al., 2020). However, comprehensive studies involving mice model using a large number of *C*. *parapsilosis* isolates are required to investigate if Y132F possess a higher virulence and if such isolates are associated with a higher mortality rate.

FTF was found in 100% and 9% of fluconazole-treated patients infected with FNS and FS *C*. *parapsilosis* isolates, respectively. Although FTF can also occur because of fluconazole tolerance (Arastehfar et al., 2020e; Berman and Krysan, 2020), none of the FS isolates showed a fluconazole-tolerant phenotype and FTF could be explained by the involvement of other mechanisms such as host- and drug-related factors (Arastehfar et al., 2020e; Arastehfar et al., 2020f; Berman and Krysan, 2020). Consistent with other studies, we found that 32% of the patients infected with FNS isolates were also azole-naïve (Arastehfar et al., 2020b), which raises the question whether such isolates have emerged through clonal expansion. Therefore, we analyzed the genetic relatedness of *C*. *parapsilosis* isolates by multilocus microsatellite typing, which allowed identification of two major clusters: one predominated by FNS and the other – by FS isolates, among which a few FNS isolates were scattered. Interestingly, all FNS *C*. *parapsilosis* isolates carrying Y132F and Y132F+G307 grouped together in genetically similar minor clusters and were mostly obtained from patients in chest diseases and pediatric surgery wards, whereas other FNS isolates carrying G458S were clonal and dispersed among FS isolates. Clonality was observed for FS *C*. *parapsilosis* and almost 37% of patients infected with such isolates were admitted to the burn ICU, which is similar to our previous observations. These data are important to consider, since some studies have focused on the genetic relatedness of FNS *C*. *parapsilosis* assuming that Y132F isolates are mostly associated with persistent clonal outbreaks, (Choi et al., 2018) while here we have shown that both FNS and FS *C*. *parapsilosis* isolates formed clonal clusters and consequently caused outbreak. The observation of expanding genetically similar and/or clonal ones is similar to what have been observed for the multidrug-resistant *Candida auris*, where cross-transmission among several patients were recorded during the course of infection (Al Maani et al., 2019; Mohsin et al., 2020). Our findings emphasize the continuous expansion of clonal FNS and FS *C*. *parapsilosis* outbreaks in EUH, which requires prompt implementation of infection control strategies and establishment of antifungal stewardship to prevent further spread and emergence of *C*. *parapsilosis* in this hospital. Of note, the lack of AMB resistance in our huge collection of *C*. *parapsilosis* isolates collected over 14 years and its well-tolerability among neonates and children is in line with the *in-vivo* efficacy data obtained from *G*. *mellonella* where the larvae were infected with MDR and FNS *C. parapsilosis* blood isolates showed excellent efficacy when AMB compared with fluconazole (Binder et al., 2020).

The major limitations of the current study are the lack of expression analysis of efflux pumps (*CDR1* and *MDR1*) and *ERG11* in FNS compared to FS *C*. *parapsilosis* isolates and their association with gain-of-function mutations occurring in their transcription factors, namely *TAC1*, *MRR1*, and *UPC2*. Moreover, the continual clonal expansion of FNS and FS *C*. *parapsilosis* isolates in EUH requires the environmental screening to find the source of infection, which will be addressed in the future.

## Data Availability Statement

The datasets presented in this study can be found in online repositories. The sequences of ERG11, FKS1-HS1, FKS1-HS2 obtained in this study were deposited in the GenBank database under the following accession numbers (MW013584–MW013641), (MW013700–MW013757), and (MW013642–MW013699), respectively.

## Ethics Statement

This study was approved by the ethical committee of EUH (ethical approval number: 20-2T/30). Written informed consent to participate in this study was provided by the participants’ legal guardian/next of kin.

## Author Contributions

Conceptualization, AA, CL-F, DP, and SH-P. Methodology, AA, CL-F, SH-P, FD, WP, AH, WF, WL, ZŞ-B, and DM. Software, AA, SH-P, FD, and AH. Validation, AA, SH-P, FD, WP, DP, and CL-F. Formal analysis, AA and SH-P. Investigation, AA, CL-F, FD, WP, AH, WF, WL, ZŞ-B, and DM. Resources, WF, WL, ZŞ-B, DM, SH-P, MI, and WP. Data curation, AA, SH-P, FD, and AH. Writing – original draft preparation draft, AA. Writing – review & editing, All co-authors. Visualization, AA, AH, and JA. Supervision, AA, SH-P, DP, and WP. Project administration, AA. Funding acquisition, DP, WP, and SH-P. All authors contributed to the article and approved the submitted version.

## Funding

This work was supported by the Major National R&D Projects of the National Health Department [2018ZX10101003], National Natural Science Foundation of China [31770161], Shanghai Science and Technology Committee [17DZ2272900 and 14495800500], Shanghai Municipal Commission of Health and Family Planning [2017ZZ01024-001], Shanghai Sailing Program [19YF1448000], and the Chinese Academy of Engineering [2019-XY-33].

## Conflict of Interest

Author AH was employed by company Biotechvana.

The remaining authors declare that the research was conducted in the absence of any commercial or financial relationships that could be construed as a potential conflict of interest.
